# Performance and Mechanisms of Sulfidated Nanoscale Zero-Valent Iron Materials for Toxic TCE Removal from the Groundwater

**DOI:** 10.3390/ijerph19106299

**Published:** 2022-05-22

**Authors:** Yue Lang, Yanan Yu, Hongtao Zou, Jiexu Ye, Shihan Zhang

**Affiliations:** 1 College of Land and Environment, Shenyang Agricultural University, Shenyang 110866, China; langyue@sjzu.edu.cn; 2 Key Laboratory of Microbial Technology for Industrial Pollution Control of Zhejiang Province, College of Environment, Zhejiang University of Technology, Hangzhou 310014, China; 1112127014@zjut.edu.cn (Y.Y.); yejiexu@zjut.edu.cn (J.Y.); shihanzhang@zjut.edu.cn (S.Z.)

**Keywords:** S-nZVI, sulfidation, trichloroethylene, pathway, groundwater safety

## Abstract

Trichloroethylene (TCE) is one of the most widely distributed pollutants in groundwater and poses serious risks to the environment and human health. In this study, sulfidated nanoscale zero-valent iron (S-nZVI) materials with different Fe/S molar ratios were synthesized by one-step methods. These materials degraded TCE in groundwater and followed a pathway that did not involve the production of toxic byproducts such as dichloroethenes (DCEs) and vinyl chloride (VC). The effects of sulfur content on TCE dechlorination by S-nZVI were thoroughly investigated in terms of TCE-removal efficiency, H_2_ evolution, and reaction rate. X-ray diffraction (XRD) and X-ray Photoelectron Spectroscopy (XPS) characterizations confirmed Fe(0) levels in S-nZVI were larger than for zero-valent iron (nZVI). An Fe/S molar ratio of 10 provided the highest TCE-removal efficiencies. Compared with nZVI, the 24-h TCE removal efficiencies of S-nZVI (Fe/S = 10) increased from 30.2% to 92.6%, and the Fe(0) consumed during a side-reaction of H_2_ evolution dropped from 77.0% to 12.8%. This indicated the incorporation of sulfur effectively inhibited H_2_ evolution and allowed more Fe(0) to react with TCE. Moreover, the pseudo-first-order kinetic rate constants of S-nZVI materials increased by up to 485% compared to nZVI. In addition, a TCE degradation was proposed based on the variation of detected degradation products. Noting that acetylene, ethylene, and ethane were detected rather than DCEs and VC confirmed that TCE degradation followed β-elimination with acetylene as the intermediate. These results demonstrated that sulfide modification significantly enhanced nZVI performance for TCE degradation, minimized toxic-byproduct formation, and mitigated health risks. This work provides some insight into the remediation of chlorinated-organic-compound-contaminated groundwater and protection from secondary pollution during remediation by adjusting the degradation pathway.

## 1. Introduction

Chlorinated hydrocarbons are common industrial solvents. Accidental spills and improper disposal result in these organic compounds entering the soil, drinking water, and groundwater [1,2,3,4,5]. Trichloroethene (TCE) is an intensively detected chlorinated hydrocarbon in groundwater [6,7]. It readily migrates, is persistent in abiotic and biotic degradation, and poses serious threats to groundwater [8,9,10,11,12]. TCE poses a significant carcinogenic risk to human health by all routes of exposure [13] and is classified as “carcinogenic to humans” by the International Agency for Research on Cancer [14]. TCE exposure is associated with kidney cancer, liver cancer, and lymphatic-system cancer. Groundwater serves as a primary source of drinking water in China, which underscores the importance of developing an environmentally friendly technology to remediate the TCE-contaminated groundwater in order to mitigate its health risks.

Methods such as adsorption, oxidation/reduction, and biologically reductive dechlorination have been used to eliminate TCE in groundwater [2,15,16]. Among these methods, nanoscale zero-valent iron (nZVI) is promising for the in situ chemical remediation of TCE-contaminated groundwater, readily reacting and dechlorinating TCE into non-toxic end products [17]. However, nZVI readily reacts with dissolved oxygen, water, and other groundwater substrates to form iron oxides and/or hydroxides. The continuously thickened iron oxide or hydroxide shell passivates the nZVI surface, which decreases the electron-transfer efficiency between Fe(0) and the pollutants in the inner core, and decreases nZVI activity [18,19,20,21,22]. Furthermore, nZVI shows relatively poor selectivity for TCE reduction over water in an aqueous solution [23].

In response to these problems, many groups have reported their research regarding ZVI modification to improve its reactivity, selectivity, and longevity. A few recent studies have reported that sulfidation could improve nZVI performance in the reduction of chlorinated contaminants. During sulfidation, FeS forms on the nZVI surface, thereby enhancing electron transfer between Fe(0) and chlorinated hydrocarbons and restraining hydrogen (H_2_) evolution between Fe(0) and water [24,25,26]. Kim et al. reported that ‘Fe/FeS’ nanoparticles, synthesized via dithionite addition, enhanced TCE removal as compared to nZVI [27]. Rajajayavel and Ghoshal reported that sulfidation of nZVI facilitated TCE dechlorination and suggested the Fe/S molar ratio played a key role in the reactivity of sulfidated nZVI (S-nZVI) [28]. Han and Yan reported that enhanced TCE dechlorination rates with increasing sulfur levels were only significant at low sulfur loadings, which differed from the findings of Rajajayavel and Ghoshal [23,28]. Thus, although incorporating sulfur into nZVI has been demonstrated, S-nZVI needs additional refinement for better TCE dechlorination, and the critical variables such as sulfur content and speciation need a systematic investigation. Moreover, the abiotic TCE-degradation pathway typically follows one of three pathways such as hydrogenolysis, α-elimination, and β-elimination [17]. Among them, β-elimination is promising since it produces acetylene as an intermediate rather than highly toxic DCEs and VC [28]. Therefore, in-depth research on the pathway of TCE dechlorination is specifically needed for S-nZVI.

In this study, S-nZVI materials with different Fe/S molar ratios were prepared using sodium dithionite as the sulfidation agent. The effects of Fe/S molar ratios on the morphology and structure were characterized by scanning electron microscopy (SEM), Brunner–Emmet–Teller measurements (BET), X-ray diffraction (XRD), and X-ray photoelectron spectroscopy (XPS). The reactivities of different S-nZVI materials and bare nZVI to TCE and water were assessed with respect to TCE-removal efficiency, H_2_ evolution, and the kinetics of TCE degradation. Furthermore, dechlorination products and pathways were identified in order to gain insight into the mechanism of S-nZVI for TCE degradation and the mitigation of health risks. This work provides some guidance to develop green and environmentally friendly technologies to remediate chlorinated-organic-compound-contaminated groundwaters and protect them from secondary pollution and potential health risks.

## 2. Materials and Methods

### 2.1. Reagents

FeSO_4_·7H_2_O (>99%, Aanalytical Reagent), ethanol (99.8%, Guaranteed Reagent) and NaOH (95%, Analytical Reagent) were purchased from Rhawn (Shanghai, China). NaBH_4_ (98%, Analytical Reagent) was purchased from Sinopharm Chemical Reagents Ltd., (Shanghai, China). TCE (C_2_HCl_3_, >99.5%, Guaranteed Reagent) and Na_2_S·9 H_2_O (>98.0%, Analytical Reagent) were purchased from Aladdin (Shanghai, China). N_2_ (purity 99.99%), air (purity 99.99%), Ar (purity 99.99%) and H_2_ (purity 99.99%) were purchased from Hangzhou Jingong Special Gas Co., Ltd. (Hangzhou, China). H_2_ standard gas (10%) and Alkane olefin standard gas (purity 99.99%) were purchased from Dalian Date Gas Co., Ltd. (Dalian, China).

### 2.2. Preparation of nZVI

A total of 4.9643 g of FeSO_4_·7H_2_O was dissolved in 50 mL deoxygenated water and transferred into a 250 mL three-necked flask. Absolute ethanol (20 mL) was added and stirred to dilute the dispersed solute and make the synthesized iron particles smaller. NaBH_4_ (2.0266 g) (excess Fe^2+^:BH_4_^−^ = 1:3) was dissolved in 20 mL of 0.1% NaOH solution (inhibiting NaBH_4_ hydrolysis). The mixture was added dropwise using a constant-pressure funnel to a three-neck flask at 2 drops/s and stirred at 1000 rpm. After the dropwise addition was completed, stirring was continued for 0.5 h. Then, the nZVI particle was separated from the suspension using a magnet, and washed with deoxygenated deionized water and pure ethanol three times. After that, the material was sequentially dried in an argon-filled glove box for 24 h and aged in the transition chamber of the argon-filled glove box for 8 h. Finally, it was sealed and stored in the anaerobic glove box. All the reactant solutions used in the experiment were stripped with N_2_ for at least 0.5 h to remove the dissolved oxygen.

### 2.3. One-Step Preparation of S-nZVI

A total of 3.723 g of FeSO_4_·7H_2_O was added into 110 mL of deoxygenated water and stirred for 0.5 h under N_2_ atmosphere in a 250 mL three-necked flask. Then, 7.5 mL of aq. NaOH (1 M) was added dropwise into the FeSO_4_ solution at a rate of 1 drop/s while stirring. A total of 1.0165 g of NaBH_4_ was dissolved into 40 mL of deoxygenated water, and a certain amount of 0.1 M Na_2_S·9H_2_O solution was added in order to prepare a synthesis mixture. After that, the mixture of NaBH_4_ and Na_2_S solution were introduced into the three-necked flask at a rate of 0.2 mL/s by a peristaltic pump, followed by 0.5 h of mechanically stirring under an atmosphere of N_2_. The dosages of Na_2_S·9H_2_O solution in the synthesis mixture were 52.23, 26.12, 17.41 and 13.06 mL to achieve the Fe/S molar ratios of 2.5, 5, 7.5 and 10, respectively. The resulting S-nZVI suspension was washed at least three times with the deoxygenated deionized water and pure ethanol, and dried in an argon-filled glove box for 24 h. Finally, the obtained S-nZVI particles were placed in the transition chamber of the anaerobic glove box for 8 h of aging, and then sealed and stored in the argon-filled glove box. All the reactant solutions used in the experiment were purged with N_2_ for at least 0.5 h to remove the dissolved oxygen.

### 2.4. TCE Degradation and H_2_ Evolution

Inside an anaerobic glove box, 0.026 g of S-nZVI or nZVI was added to a 52 mL crimp-top vial containing 26 mL of a deoxygenated 4-(2-hydroxyethyl)-1-piperazine ethane sulfonic acid (HEPEs) buffer (50 mM, pH = 7), and sealed with an aluminum cover and a polytetrafluoroethylene (PTFE) septum. Outside the glove box, 15 μL of a 0.136 M TCE stock solution was injected to ensure an initial TCE concentration of 10 ppm. Finally, the crimp-top vial was placed on a constant-temperature shaker and maintained at 30 °C. At certain intervals, 100 μL of headspace gas in the bottle was extracted with a micro-sampling needle; the TCE concentration, H_2_ concentration, and the TCE-degradation products were measured by GC. The solution pH in the bottle was measured after the reaction.

### 2.5. Characterization

A physical adsorption instrument (Mike 2460 type, Norcross, GA, USA) measured the adsorption isotherm of the material on N_2_ at 77 K using N_2_ as the adsorbate to calculate the specific surface area and pore size of the material. The samples were degassed at 120 °C for 6 h before testing, and the specific BET surface area of the tested material was determined based on the N_2_ adsorption and desorption of BJH models at 77 K. The surface morphology of the material was analyzed by SEM (Zeiss Sigma 300, Oberkochen, Germany). The surface composition of the material was analyzed using XPS (K-AlpHa, Thermo Scientific, Waltham, MA, USA). The initial binding energy of the XPS spectrum was corrected based on the C1s peak at 284.8 eV, and the energy spectrum was analyzed using XPS-peak fit software (V.4.1, USA). The crystal type of the material was qualitatively analyzed by XRD (X’Pert’3 Powder, Panalytical, Almelo, The Netherlands). The test conditions of the instrument were as follows: the X-ray source was Cu target Kα rays (λ = 0.154056 nm), with a scanning range of 10–80° at a scanning rate of 5° min^−1^.

### 2.6. Kinetic Modelings

The degradation of TCE followed the pseudo-first-order reaction kinetics (Equation (1))

(1) $${y=\mathrm{Ae}}^{{-k}_{\mathrm{sa}}t}$$

where *k_sa_* (L/(m^2^·h)) refers to the pseudo-first-order reaction-rate constant, *y* (mg/L) represents the TCE concentration of headspace sampling in the crimp-top vial, *t* (h) represents the reaction time, and *A* (mg/L) represents the initial TCE concentration.

### 2.7. Analytical Methods

Concentrations of TCE and its degradation products were measured by a GC (Agilent 7890B, Santa Clara, CA, USA) equipped with a flame ionization detector (FID). The capillary column was a GS-Q, 30 m long with a 0.53 mm diameter. The inlet temperature was 200 °C, the detector temperature was 230 °C, the injection volume was 100 μL, and the injection port split ratio was 10:1. The initial column oven temperature was 50 °C, maintained for 7 min, then increased to 230 °C at a heating rate of 20 °C/min for 10 min. During this process, TCE and its degradation products in the samples were separated.

The concentration of H_2_ was measured by a gas chromatograph (FULI-9790, Taizhou, China) equipped with a thermal conductive detector (TCD). A TXD-01 packed column (3 mm × 2 m, Lanzhou Institute of Chemical Physics, Chinese Academy of Sciences, Lanzhou, China) was used, with Ar as the carrier gas. The inlet temperature was 140 °C, the column oven was 60 °C, the detector temperature was 140 °C, and the injection volume was 100 µL. Since H_2_ solubility in water is negligible, H_2_ in the reaction-vial headspace was the total H_2_ produced during the reaction.

## 3. Results and Discussion

### 3.1. Materials Characterization

**Figure 1:**
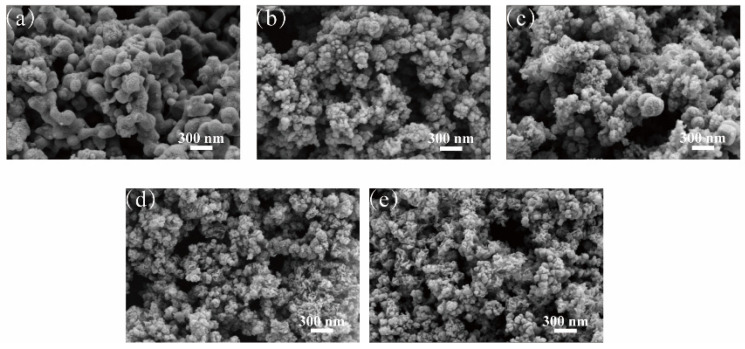
SEM images of nZVI and S-nZVI materials with different Fe/S molar ratios (**a**) nZVI; (**b**) S-nZVI (Fe/S = 10); (**c**) S-nZVI (Fe/S = 7.5); (**d**) S-nZVI (Fe/S = 5); (**e**) S-nZVI (Fe/S = 2.5).

Figure 1 shows the surface morphology of nZVI and S-nZVI materials with different Fe/S molar ratios. Figure 1a shows spherical nZVI particles with relatively uniform sizes (50–200 nm) that aggregate into chains due to electrostatic and magnetic forces. The S-nZVI contained spherical and amorphous structures as shown in Figure 1b–e. By comparing S-nZVI materials with different Fe/S ratios, the spherical structure continuously decreased, and the main structure gradually changed from a relatively regular sphere to an irregular amorphous structure as sulfur was added. These changes illustrated that sulfidation increased material dispersion and improved its specific surface area, which could be related to the formation of an FeS_x_ layer on the nZVI surface.

**Figure 2:**
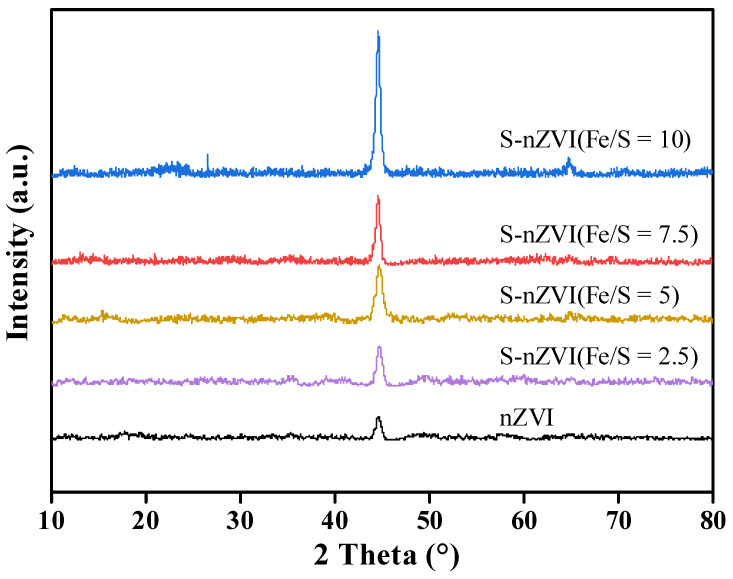
XRD spectra of nZVI and S-nZVI materials with different Fe/S molar ratios.

Figure 2 shows XRD spectra of nZVI and S-nZVI with different Fe/S ratios. The characteristic diffraction peaks at 2θ = 44.6° and 65.0° corresponded to the (110) and (200) faces of Fe(0) (PDF#00-006-0696), respectively. On the other hand, there was no obvious iron-oxide peak, which indicated that the degree of oxidation was relatively light during the preparation and preservation of the material. Additionally, S-nZVI had a strong diffraction peak at the same position, which indicated high crystallinity Fe(0) in S-nZVI, and that sulfur introduction promoted the formation of Fe(0) crystals, and the peak intensity increased with the increase of Fe/S ratio.

The pore structure and size of different materials were analyzed to explore surface-structure differences among nZVI and S-nZVI materials with different Fe/S ratios. As shown in Table 1, the specific surface area of nZVI was 8.76 m^2^/g, and the specific surface areas of S-nZVI with different Fe/S ratios (2.5, 5, 7.5, and 10) were 95.66, 53.52, 57.60, and 27.11 m^2^/g, respectively. The specific surface area and pore volume increased with added sulfur, perhaps due to the presence of FeS_x_, which inhibited material agglomeration and increased the surface roughness and surface area. For Fe/S = 5 and 2.5, the average pore size and volume both increased significantly. This might be due to increased FeS_x_; the amorphous structure became the primary structure, which resulted in larger pore size and volume. This conclusion agreed with SEM image data. These results indicated the Fe/S ratio significantly impacted the pore size and characteristics of S-nZVI as well as its reaction activity.

**Figure 3:**
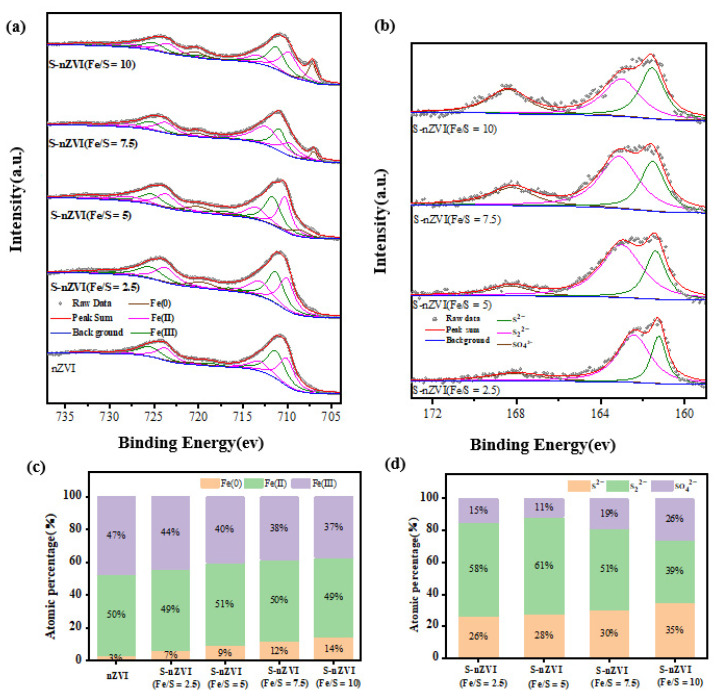
XPS spectra of nZVI and S-nZVI materials with different Fe/S ratios: (**a**) XPS spectrum of Fe2 p; (**b**) XPS spectrum of S 2p; (**c**) Fe levels in different valences; (**d**) S levels in different valences.

XPS analysis determined the elemental composition and valence information on the material surface. Fe 2p peak fitting (Figure 3a) yielded binding energies for Fe 2p 3/2, Fe(III), Fe(II), and Fe(0) at 711.0, 709.0, and 706.7 eV, respectively, while Fe 2p 1/2, Fe(III), Fe(II), and Fe(0) had binding energies of 724.8, 722.8, and 719.8 eV, respectively. Shoulder peaks for Fe(III) and Fe(II) came at 718.9 and 733.4 eV, and 714.6 and 729.5 eV, respectively. Additionally, no obvious elemental-iron peaks were observed because the XPS analysis determined the nanomaterial components on the surface. Fitting data revealed the S-nZVI surface was covered by iron sulfide, so the ZVI content was not detected. As shown in Figure 3c, the one-step sulfidation of nZVI showed a drop in the Fe(III) content with additional sulfur, while levels of Fe(II) remained steady. Peak fitting of S 2p in the material is shown in Figure 3b, in which the binding energies of monosulfide (S^2−^), disulfide (S_2_^2−^), and sulfate (SO_4_^2−^) were 161.5, 162.2, and 168.5 eV, respectively. As shown in Figure 3d, S in S-nZVI mainly existed as S^2−^, S_2_^2−^, or SO_4_^2−^. With added sulfur, S^2−^ levels decreased, and the content of S_2_^2−^ increased, possibly due to excessive sulfur (from sodium sulfide) that reduced to S_2_^2−^, and the electron-transfer utilization of FeS_2_ was much smaller than FeS [29], so the generation of excessive S_2_^2−^ reduced its electron-transfer efficiency and reduced dechlorination of S-nZVI on TCE.

### 3.2. Performance of TCE Degradation

**Figure 4:**
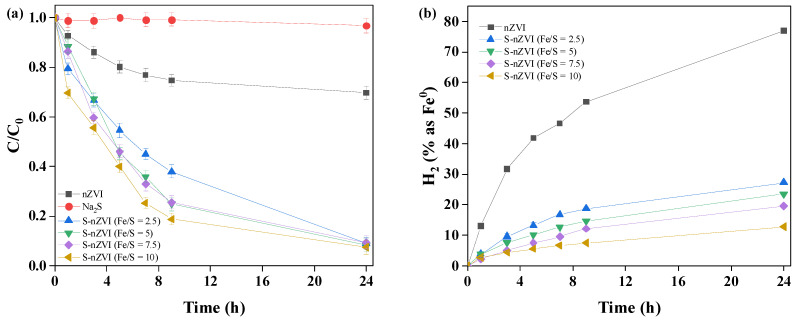
Performance of nZVI and S-nZVI materials with different Fe/S molar ratios in TCE degradation, (**a**) TCE removal; (**b**) H_2_ evolution (pH: 7, t: 30 °C; S-nZVI: 0.026 g, TCE: 10 ppm).

Figure 4 shows the performances of nZVI and S-nZVI materials with different Fe/S ratios (Fe/S = 2.5, 5, 7.5, and 10) in TCE degradation. As shown in Figure 4a, the 24 h TCE-removal efficiency of nZVI was 30.2%, while that of S-nZVI (Fe/S = 10) exceeded 92.6%, which indicated that nZVI did not degrade TCE, while the dechlorination of S-nZVI improved significantly. Meanwhile, the removal efficiency of TCE increased as the Fe/S molar ratio increased. To confirm whether Fe (not S) degraded TCE in S-nZVI, Na_2_S was mixed with TCE to monitor its degradation effect. As shown in Figure 4a, Na_2_S did not degrade TCE. A quantitative test of H_2_ in the crimp-top vial was performed (Figure 4b) to explore how much Fe(0) was used for H_2_ evolution. That test indicated that the side reaction of H_2_ evolution consumed 77.0% of the initial Fe(0) in nZVI. However, only 12.8% of the initial Fe(0) was consumed for H_2_ evolution in the S-nZVI at an Fe/S ratio of 10. This indicated that sulfidation greatly inhibited side reactions so that a large amount of Fe(0) could react fully with TCE. Some researchers reported that H_2_ evolution mainly occurred in the iron oxide layer rather than the FeS_x_ layer, while the main reaction site for TCE occurred in the FeS_x_ layer [30,31]. Since the FeS_x_ layer had strong electrical conductivity [32], electron transfer from the surface of the FeS_x_ layer was used for TCE degradation. This was the reason S-nZVI simultaneously suppressed H_2_ evolution and promoted TCE degradation.

### 3.3. Kinetics of TCE Degradation

**Figure 5:**
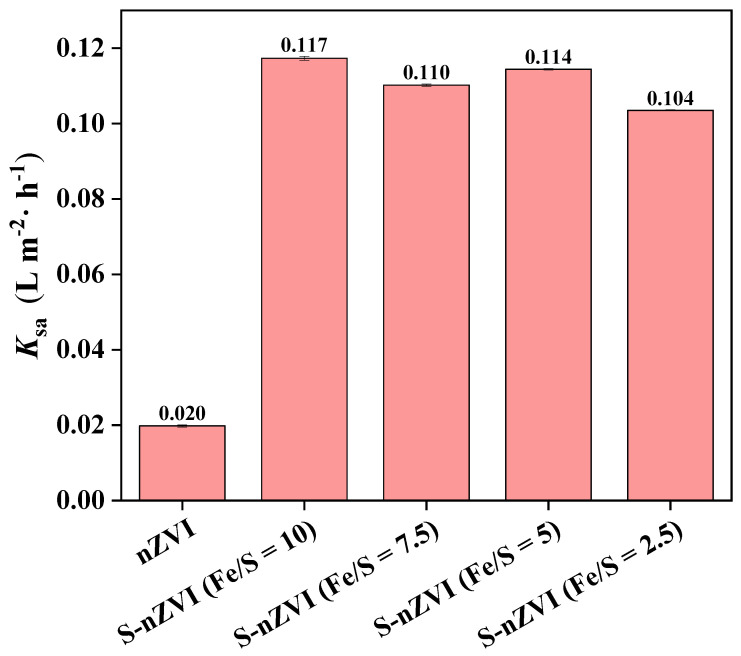
Reaction rates of TCE degradation by nZVI and S-nZVI materials with different Fe/S molar ratios. (pH: 7, t: 30 °C; nZVI and S-nZVI: 0.026 g, TCE: 10 ppm).

An evaluation of degradation rate constants for nZVI and S-nZVI materials was conducted and is shown in Figure 5. Rate constants were obtained by fitting the 24 h removal-efficiency curve for TCE using pseudo-first-order kinetics. As shown in Figure 5, the incorporation of sulfur into nZVI greatly increased the degradation rate constant, which was likely due to the FeS shell of S-nZVI improving electron-transfer efficiency. The degradation activity of S-nZVI maximized for Fe/S = 10, with a rate constant of 0.117 L/(m^2^·h). However, when the Fe/S molar ratio varied between 2.5–10, the rate constant for S-nZVI materials changed slightly. This may be related to excess Fe(0) in the S-nZVI system, where the initial TCE concentration was as low as 10 ppm.

### 3.4. Mechanism of TCE Degradation

**Figure 6:**
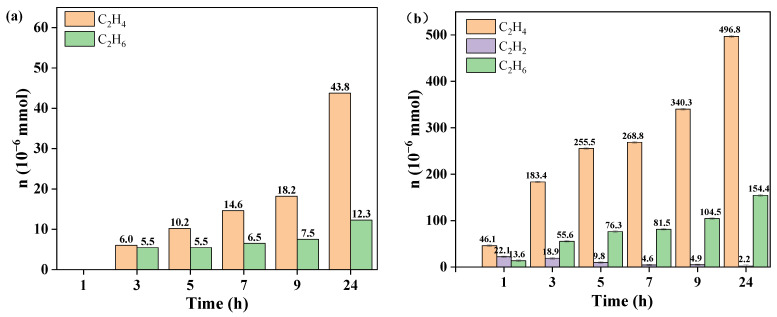
Gas phase production during TCE degradation with: (**a**) neat nZVI; and (**b**) S-nZVI with a Fe/S ratio of 10 (pH: 7, T: 30 °C; nZVI and S-nZVI: 0.026 g).

As shown in Figure 6, toxic products such as DCEs and VC were not detected in either neat nZVI or S-nZVI. Moreover, both materials primarily produced ethylene, which confirmed that TCE degradation followed a β-elimination mechanistic pathway with acetylene as the intermediate. In this work, primary products of TCE degradation such as acetylene, ethylene, and ethane were measured and are shown in Figure 6. Compared with the neat nZVI, a small amount of acetylene was detected with the S-nZVI, which indicated that sulfidated modification of nZVI enhanced the conversion of TEC to form acetylene. Moreover, the ethylene produced using S-nZVI was 11.3 times greater than nZVI, which confirmed that sulfidated modification enhanced the reactivity of each step during TCE degradation.

**Figure 7:**
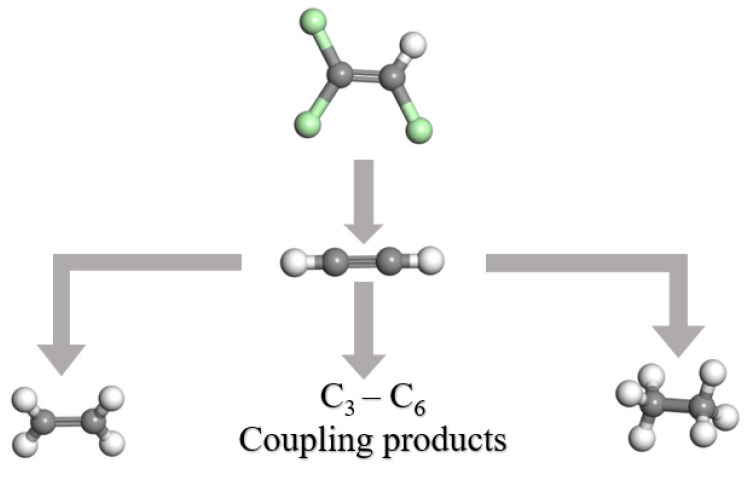
A possible mechanism for the TCE degradation with the S-nZVI.

Overall, dechlorination of TCE occurred via β-elimination through chloroacetylene, followed by hydrogenolysis of chloroacetylene to acetylene, which agreed with previous reports [23,33]. A larger dose of S-nZVI produced ethylene and ethane via hydrogenation of acetylene, and may generate C3-C6 hydrocarbons via the polymerization of acetylene (Figure 7).

## 4. Conclusions

This study found that sulfidation significantly improved TCE dechlorination via nZVI. XRD and XPS results illustrated that sulfidation caused the material to store more Fe(0), and Fe(0) increased with the decreasing sulfur ratio (Fe/S ≤ 10). The 24 h TCE-removal efficiency of S-nZVI with an Fe/S ratio of 10 increased from 30.2% to 92.6% compared to nZVI. This was attributed to a reduction in H_2_ evolution from nZVI after sulfidation. At an Fe/S molar ratio of 10, Fe(0) consumed by H_2_ evolution was only 12.8%, which suggested a large amount of reactive Fe(0) was available to react with TCE. Compared with nZVI, the pseudo-first-order kinetic rate constants of S-nZVI materials increased significantly. Additionally, the reaction of TCE degradation by S-nZVI was explored. Acetylene was the intermediate product, followed by the production of ethylene and ethane. C_3_–C_6_ hydrocarbons may be generated via the polymerization of acetylene. Overall, this work provides insights into developing green and environmentally friendly technologies to remediate chlorinated organic compound-contaminated groundwater and protect them from secondary pollution as well as potential health risks in groundwater utilization.

## Figures and Tables

**Table 1:** Pore-structure parameters of nZVI and S-nZVI materials with different Fe/S ratios.

Sample	*S*_BET_ (m^2^/g)	*D*_average_ (nm)	*V*_total_ (cm^3^/g)
nZVI	8.76	3.41	0.01
S-nZVI (Fe/S = 10)	27.11	3.82	0.05
S-nZVI (Fe/S = 7.5)	57.60	3.41	0.12
S-nZVI (Fe/S = 5)	53.52	22.17	0.31
S-nZVI (Fe/S = 2.5)	95.66	18.91	0.48

## Data Availability

The data presented in this study are available on request from the corresponding author. The data are not publicly available due to privacy.
